# Prévalence et déterminants de l'allaitement maternel poursuivi au-delà de 6 mois chez les femmes algériennes. Analyse à partir des données de l'enquête par grappes à indicateurs multiples

**DOI:** 10.48327/mtsi.v4i1.2024.499

**Published:** 2024-03-08

**Authors:** Ahcène ZEHNATI, Adel SIDI-YAKHLEF

**Affiliations:** 1 Directeur de recherche au Centre de recherche en économie appliquée pour le Développement (CREAD), Algérie et chercheur associé au Laboratoire d’économie de Dijon (LEDi), France; 2 Professeur au Département des sciences sociales, Faculté des sciences humaines et sociales, Université Abou-Bakr-Belkaid Tlemcen, Algérie

**Keywords:** Allaitement maternel, Sevrage, Prévalence, Déterminants, Enquête par grappes à indicateurs multiples, Algérie Maghreb, Afrique du Nord, Breastfeeding, Weaning, Prevalence, Determinants, Multiple Indicators Cluster Survey, Algeria, Maghreb, Northern Africa

## Abstract

**Introduction:**

L'allaitement maternel (AM) est considéré comme la meilleure alimentation pour les nourrissons et joue un rôle important dans la croissance et le développement de l'enfant. À cet égard, l'Organisation mondiale de la santé (OMS) recommande fortement l'allaitement exclusif dans les 6 premiers mois de la vie, ainsi que la poursuite de l'allaitement lors de l'introduction d'aliments sûrs et appropriés jusqu’à l’âge de 2 ans ou au-delà.

**Justification:**

Alors que la littérature existante sur l'AM dans les pays développés est foisonnante, force est de constater que dans les pays en développement à l'instar de l'Algérie, cette problématique est sous analysée. Notre étude contribue à estimer la prévalence de l'AM et à identifier les facteurs qui lui sont associés.

**Méthodes:**

Il s'agit d'une étude transversale descriptive de l'ensemble des enfants qui ont été allaités et qui figurent dans la base de données de l'enquête à indicateurs multiples (MICS) réalisée en 2019, soit 8 709 enfants, dont 4 471 garçons et 4 238 filles. Afin d'explorer les facteurs associés à la durée de l'AM nous n'avons retenu que les enfants sevrés afin de minimiser le biais concernant les enfants qui étaient toujours allaités, ce qui nous a permis d'analyser les données de 3 761 enfants, dont 1 930 garçons (51,4 %) et 1 831 filles (48,6 %). En utilisant un modèle de régression logistique, nous avons pu évaluer le rôle de différents facteurs sociodémographiques, économiques et géographiques dans le maintien de l'allaitement au-delà de 6 mois.

**Résultats:**

Le taux d'AM global était de 81,1 %; le taux d'allaitement exclusif des enfants de 0 à 6 mois s’élevait à 28,7 %. Cependant, les principaux facteurs associés à l'AM étaient le milieu de résidence, la région géographique, le statut professionnel de la mère et le quintile de richesse. Les facteurs tels que le sexe de l'enfant, le niveau d'instruction et les difficultés fonctionnelles des mères ne semblaient pas être des facteurs déterminants pour le maintien de l'allaitement après six mois.

**Discussion - Conclusion:**

La prévalence de l'allaitement est quasiment identique à celle des études locales recensées. Cependant, les déterminants du recours à l'allaitement ne sont pas toujours en phase avec nos conclusions. Cette étude revêt une grande importance dans les pays en développement tels que l'Algérie, en vue de l'adoption d'interventions préventives, notamment en matière de communication et de conseils pré et postnataux dans le cadre du projet d'allaitement.

## Abstract (en)

**Background and rationale:**

Breastfeeding is considered the best nutrition for infants and plays a significant role in a child's growth and development. In this regard, the World Health Organization (WHO) strongly recommends exclusive breastfeeding for the first 6 months of life, as well as continued breastfeeding when safe and appropriate complementary foods are introduced, up to 2 years of age or beyond. Nonetheless, breastfeeding practices remain sub-optimal in many parts of the world. In this respect, many children have shown not to be breastfed up to 6 months of age or are not breastfed during their first hours of life or are weaned too early. This allows for the use of infant formula, which can be life threatening due to poor hygiene, storage conditions or inappropriate foods. Likewise, breastfeeding rates in the Middle East and North Africa (MENA) region are lower than the global average. In Algeria, the breastfeeding rate up to 6 months of age is among the lowest in the world according to previous Multiple Indicators Cluster Surveys (MICS) reports. In addition, by the end of the sixth month, the percentage of children exclusively breastfed is less than 3% and only 23% of children receive breast milk at 22-23 months. The median duration of breast-feeding is 12 weeks. However, the socio-demographic factors associated with this sub-optimal breastfeeding practice are not explored in these reports, as studies and research conducted in this context using other data are few and mostly conducted in limited geographical areas.

**Materials and methods:**

This stands for a descriptive cross-sectional study of all children who were breastfed in the MICS database, carried out in 2019, 8 709 children, including 4 471 boys and 4 238 girls, registered in the database. To explore factors associated with the duration of breastfeeding, we only included weaned children to minimize bias related to children who were still breastfeeding. This allowed to analyze data from 3 761 children, including 1 930 boys (5.4%) and 1,831 girls (48.6%). Through using a logistic regression model, we could attain the assessment of the role of different socio-demographic, economic and geographical factors in the maintenance of breastfeeding beyond 6 months.

**Results:**

The prevalence of breastfeeding is 81.1%; the rate of exclusive breastfeeding of children aged 0-6 months is 28.7%. The factors associated with breastfeeding beyond six months are area of residence, mother's occupational status and wealth quintile. In fact, rural area appears to be a factor favoring the continuation of breastfeeding beyond six months, compared with urban area (OR = 1.29; CI [1.032-1.369]). Mothers living in the Eastern Highlands geographic region appear to be 1.56 times more likely to maintain breastfeeding for more than six months compared to those living in the Southern region (OR = 1.56; CI [1.123 – 1.677]). Children of non-working mothers are almost 1.5 times more likely to be breastfed after 6 months than those of working mothers (OR = 1.489; CI [1.107 – 1.947]). Parents belonging to the “richest” wealth quintiles appear to increase the chance of continued breastfeeding after 6 months with a 1.24-fold increase compared to those belonging to the “poorest” quintile (OR = 1.24; CI [1.086 - 1.812]). Factors such as the child's gender, maternal education level, and functional difficulties do not appear to be determining factors for the continuation of breastfeeding after six months.

**Discussion and conclusion:**

The prevalence and factors associated with the initiation and continuation of breastfeeding vary from one country to another. Notwithstanding the numerous studies to better comprehend mothers’ breastfeeding behaviour and various initiatives for promotion thereof, many countries have low rates. In consequence, the downward trend in breastfeeding is directly linked to advances in the production and marketing of industrial milks, the lack of information and awareness among mothers and the lack of training of health professionals. In this respect, it is highly recommended to encourage behavioural changes, to improve communication about the duration of breastfeeding, to increase the use of postnatal counselling and training of paramedical staff according to the recommendations with support for mothers.

This study is of great interest in developing countries like Algeria, in order to adopt preventive interventions, and to organise communication and pre- and post-natal counselling in the breastfeeding project. Clearly, this study should be enhanced with supplementary qualitative studies concerning the factors contributing to early discontinuation of breastfeeding.

## Introduction

En 2015, l'Organisation des Nations unies (ONU) a adopté un agenda portant sur l'atteinte des Objectifs de développement durable (ODD) à l'horizon 2030. L'allaitement maternel (AM) est essentiel à la réalisation d'un bon nombre de ces objectifs. Il contribue à améliorer la nutrition (ODD 2), prévenir la mortalité infantile et réduire le risque de maladies non transmissibles (ODD 3) et favoriser le développement cognitif et l’éducation (ODD 4) [18]. De plus, l'Organisation mondiale de la santé (OMS) et le Fonds des Nations unies pour l'enfance (UNICEF) recommandent depuis 2001 l'AM exclusif pendant les 6 premiers mois de vie, ainsi que la poursuite de l'allaitement lors de l'introduction d'aliments sûrs et appropriés jusqu’à l’âge de 2 ans ou au-delà [13].

Cependant, malgré les avantages cruciaux de l'AM mentionnés ci-dessus, les pratiques d'AM restent sous-optimales dans de nombreuses régions du monde y compris dans les pays à haut niveau de ressources. À cet égard, de nombreux enfants ne sont pas allaités jusqu’à l’âge de 6 mois, ne sont pas allaités au cours des premières heures de leur vie, ou sont sevrés trop tôt [18]. Cela entraîne l'utilisation de lait maternisé, ce qui peut être dangereux en raison de mauvaises conditions d'hygiène, de stockage ou d'aliments inappropriés. De même, les taux d'AM dans la région du Moyen-Orient et de l'Afrique du Nord (MENA) sont inférieurs à la moyenne mondiale. Une méta-analyse de 19 études menées dans les pays de la MENA a montré que 34,3 % des nouveau-nés commençaient à être allaités dans la première heure suivant la naissance, mais seulement 20,5 % étaient exclusivement allaités pendant les 6 premiers mois [2].

En Algérie, le taux d'allaitement maternel jusqu’à l’âge de 6 mois est parmi les plus bas au monde selon les rapports des enquêtes par grappes à indicateurs multiples menées (en anglais MICS) jusqu’à présent [19]. De plus, à la fin du sixième mois, moins de 3 % des enfants sont exclusivement allaités et seuls 23 % des enfants reçoivent du lait maternel à 22-23 mois. La durée médiane de l'AM est de 12 semaines [9]. Cependant, les facteurs socio-démographiques associés à cette pratique d'AM sous-optimale ne sont pas explorés dans ces rapports. Les études menées à partir des données d'enquêtes nationales sont inexistantes. Celles consacrées à cette thématique ne sont pas nombreuses et sont conduites le plus souvent dans des territoires géographiques limités [1, 3, 4, 7, 9, 10, 11, 12].

L'objectif de cet article est d'explorer la question de l'AM en Algérie, qui reste encore sous analysée notamment à l’échelle nationale. La plupart des études sur ce sujet sont conduites dans des contextes très localisés. Pour ce faire, nous avons exploité les données issues de la dernière enquête sur la santé de la mère et de l'enfant (MICS-6) réalisée en 2019 [19]. Les résultats issus de cette contribution permettront aux responsables en charge de la planification familiale de disposer d'outils d'aide à la décision pour mieux orienter les politiques publiques en la matière.

## Matériels et méthodes

Il s'agit d'une étude descriptive transversale nichée dans la 6^e^ enquête par grappes à indicateurs multiples [*Multiple Indicator Cluster Survey,* MICS] réalisée en Algérie en 2019. Rappelons que le programme international d'enquêtes auprès des ménages, connu sous le nom d'enquête à indicateurs multiples (MICS), a été élaboré par l'UNICEF. Son objectif est de recueillir des estimations statistiquement fiables et comparables au niveau international des indicateurs clés utilisés pour évaluer la situation des enfants et des femmes dans les domaines de la santé, de l’éducation, de la protection des enfants et du VIH/Sida. Ces données jouent un rôle crucial dans l'orientation des politiques et l’évaluation des progrès envers les Objectifs de développement durable (ODD). Cette enquête permet d'appuyer les pays à disposer des données nécessaires pour le suivi des indicateurs clés relatifs à la situation des femmes et des enfants. Depuis sa création dans les années 1990, le MICS a conduit 368 enquêtes dans 120 pays. L'Algérie fait partie des 60 pays engagés dans l'initiative MICS depuis son lancement en 1990 en réalisant successivement cinq éditions.

Notre sujet d’étude a inclus tous les enfants allaités recensés dans la base de données de la 6^e^ enquête par grappes à indicateurs multiples réalisée en 2019 par la direction de la Population du ministère de la Santé, de la population et de la réforme hospitalière dans le cadre du programme mondial des enquêtes MICS. Elle est conduite avec l'appui financier et technique du Fonds des Nations unies pour l'enfance (UNICEF) et une contribution financière du Fonds des Nations unies pour la population (UNFPA). Le nombre d'indicateurs couverts par la MICS 6 s’élève à 200 dont 26 indicateurs participant directement à la mesure de 10 des 17 Objectifs du développement durable (ODD) pour l'Algérie. Sur le plan national et international, la MICS constitue une source d'information importante pour le suivi des progrès vers les ODD.

L'enquête MICS 6 a porté sur un échantillon de 31 325 ménages répartis en 7 zones géographiques dénommées espaces de programmation territoriale (EPT), ce qui permet de représenter statistiquement toute la population algérienne au niveau national et au niveau de ces territoires. Les détails de l’échantillonnage sont décrits dans le rapport de l'enquête disponible sur le site officiel de l'UNICEF [19]. Les données qui nous ont permis d'analyser la durée d'allaitement sont issues du module relatif aux « mères en charge d'enfants de moins de 5 ans ». Pour déterminer la catégorie de durée d'allaitement, nous avons pris en considération toutes les mères ayant répondu avoir allaité et sevré leurs enfants : « L'enfant a-t-il déjà été allaité ? A quel âge l'enfant a été sevré ? ». Sur cette base, nous avons construit une variable composée de quatre catégories de durée d'allaitement « moins de 3 mois », « entre 3 et 6 mois », « entre 6 et 12 mois » et enfin « plus de 12 mois ».

Afin de mieux cerner les facteurs associés à la durée d'allaitement, nous avons retenu comme variables explicatives les caractéristiques socio-démographiques, économiques et géographiques telles que le sexe de l'enfant, le niveau d’éducation de la mère, le statut professionnel de la mère, le niveau socio-économique exprimé en quintile de richesse, le milieu de résidence (urbain/rural) et la zone géographique, exprimée par l'espace de programmation territoriale [8].

Le traitement statistique des données a été réalisé avec le logiciel statistique SPSS version 25. D'abord, le test d'indépendance du chi-deux (χ^2^) a été utilisé pour effectuer les comparaisons de fréquences. Ensuite, les associations entre le statut d'allaitement et les variables prédictives potentielles ont été mesurées à l'aide des tests de régression logistique. Pour cette analyse, nous avons regroupé les quatre durées d'allaitement en deux pour construire une autre variable dépendante binaire « moins de 6 mois » et « plus de six mois ». L'ampleur de l'association a été évaluée au moyen d'odds ratios (OR) et de leur intervalle de confiance IC) à 95 %. Des seuils de signification (p) inférieurs à 0,05 ont été retenus.

## Résultats

Notre étude a porté sur 8 709 enfants, comprenant 4 471 garçons (51,4 %) et 4 238 filles (48,6 %), identifiés dans la base de données. Le taux global d'allaitement maternel a été estimé à 81,1 %. Il n'y avait pas de différence significative entre les taux d'allaitement selon le sexe (81,7 % vs 80,5 %). En ce qui concerne l'AM exclusif jusqu’à 6 mois, le taux était de 28,7 %.

Afin d'explorer les facteurs associés à la durée de l'AM, nous n'avons retenu que les enfants sevrés au jour de l'enquête, afin de minimiser le biais concernant les enfants qui étaient toujours allaités, ce qui nous a amenés à analyser les données de 3 761 enfants, dont 1 930 garçons (51,4 %) et 1 831 filles (48,6 %) (Tableau I). Le taux d'AM global s’établit à 81,1 %; le taux d'AM exclusif des enfants âgés entre 0 et 6 mois est de 28,7 %. Le tableau II présente le statut d'allaitement selon le milieu de résidence. Les enfants allaités pendant une durée inférieure à 6 mois sont à 62,3 % d'origine urbaine contre 37,7 % issus du milieu rural. Quant aux enfants ayant bénéficié d'une durée d'allaitement supérieure à 6 mois, 58,6 % d'entre eux résident en milieu urbain et 41,4 % sont d'origine rurale.

**Tableau I:** Répartition des enfants sevrés au jour de l'enquête selon le sexe et le statut d'allaitement (MICS6, Algérie, 2019) Distribution of weaned children on the day of the survey by sex and age group (MICS6, Algeria, 2019)

	Effectif en fonction du statut d'allaitement		Total
Sexe de l'enfant	Moins de 6 mois	6 mois et plus	
garçons*	1 385 (51,4 %)	545 (51 %)	1 930 (51,3 %)
filles*	1 307 (48,6 %)	524 (49 %)	1 831 (48,7 %)
**Total**	**2 692 (100 %)**	**1 069 (100 %)**	**3 761 (100 %)**

**Tableau II:** Répartition des enfants sevrés au jour de l'enquête selon la tranche d’âge et le milieu de résidence (MICS6, Algérie, 2019) Distribution of weaned children on the day of the survey by age group and place of residence (MICS6, Algeria, 2019)

	Statut d'allaitement		Total
Milieu de résidence	Moins de 6 mois	6 mois et plus	
urbain (effectif et % dans statut allaités)	1 676 (62,3 %)	626 (58,6 %)	2 302 (61,2 %)
rural (effectif et % dans statut allaités)	1 016 (37,7 %)	443 (41,4 %)	1 459 (38,8 %)
**Total**	**1 069 (100 %)**	**3 761 (100 %)**	

Le tableau III illustre la répartition des périodes d'allaitement selon les facteurs socioéconomiques et géographiques étudiés.

**Tableau III:** Répartition du statut d'allaitement selon les facteurs étudiés Distribution of breastfeeding status according to the studied factors

**Variables**	**3 mois et moins**	**Plus de 3 mois à 6 mois**	**Plus de 6 mois à 12 mois**	**Plus de 12 mois**	**Degré de significativité**
	**Effectif (%)**	**Effectif (%)**	**Effectif (%)**	**Effectif (%)**	
**Sexe de l'enfant**					
garçons	1 224 (63,4)	322 (16,7)	204 (10,6)	180 (9,3)	NS
filles	1 123 (61,3)	338 (18,5)	199 (10,9)	171 (9,3)	
**Zone d'habitat**					
urbain	1 467 (63,7)	407 (17,7)	236 (10,3)	192 (8,3)	< 0,05
rural	880 (60,3)	253 (17,3)	167 (11,4)	159 (10,9)	
**Espace de programmation territoriale (EPT)**					
EPT 1 : Nord-Centre	278 (58,9)	105 (22,2)	51(10,8)	38 (8,1)	< 0,001
EPT 2 : Nord-Est	375 (66,7)	83(14,5)	55 (10,3)	56 (8,5)	
EPT 3 : Nord-Ouest	252 (64,0)	67(17,0)	41(10,4)	34 (8,6)	
EPT 4 : Hauts Plateaux-Centre	448 (63,1)	95 (13,4)	89 (12,5)	78 (11,0)	
EPT 5 : Hauts Plateaux-Est	321 (57,1)	103 (18,3)	68 (12,1)	70 (12,5)	
EPT 6 : Hauts Plateaux-Ouest	297 (60,6)	125 (25,5)	41 (8,4)	27 (5,5)	
EPT 7 : Sud	375 (65,9)	83 (14,6)	55 (9,8)	56 (9,7)	
**Niveau d'instruction de la mère**					
sans instruction	313 (62,1)	85 (16,9)	55 (10,9)	51 (10,1)	NS
primaire	318 (59,3)	100 (18,7	65 (12,1)	53 (9,9)	
moyen	797 (65,0)	201 (16,4)	125 (10,2)	104 (8,5)	
secondaire	519 (61,2)	160 (18,9)	95 (11,2)	74 (8,7)	
supérieur	400 (61,9)	114 (17,6)	63 (9,8)	69 (10,7)	
**Difficulté fonctionnelle**					
à une difficulté fonctionnelle	77 (66,4)	13 (11,2)	9 (7,8)	17 (14,7)	NS
pas de difficulté fonctionnelle	2248 (62,2)	645 (17,8)	391 (10,8)	333 (9,2)	
**Statut professionnel de la mère**					
à une profession	237 (62,9)	77 (20,4)	31(8,2)	32 (8,5)	< 0,05
sans profession	2034 (60,1)	580 (17,1)	378 (11,2)	392 (11,6)	
**Quintiles de l'indice de richesse**					
le plus pauvre	555 (61,4)	165 (18,3)	106 (11,7)	78 (8,6)	< 0,01
le second	560 (64,2)	150 (17,2)	95 (10,9)	67 (7,7)	
le moyen	500 (61,7)	127 (15,7)	91 (11,2)	92 (11,4)	
le quatrième	448 (66,7)	108 (16,1)	61 (9,1)	55 (8,2)	
le plus riche	284 (56,5)	110 (21,9)	50 (9,9)	59 (11.7)	

Nos résultats montrent que 62,4 % des enfants ont été allaités moins de 3 mois, 17,6 % entre 3 et 6 mois, 10,1 % entre 6 et 12 mois, enfin 9,3 % plus de 12 mois. Le test d'indépendance de χ^2^ montre l'existence d'une association significative entre le statut d'allaitement et la zone de résidence, avec une proportion plus élevée de l'allaitement « + de 12 mois » chez les enfants résidant dans les zones rurales. Une association positive a également été relevée entre les régions géographiques et le statut d'allaitement. Ceci est imputable au taux d'allaitement élevé entre 3 et 6 mois dans les régions Nord-Centre et les Hauts Plateaux-Ouest et + de 12 mois dans les régions Hauts Plateaux-Centre et Est. Une corrélation significative a été également observée entre le statut professionnel de la mère et le statut d'allaitement liée principalement à la proportion élevée des mères sans travail dont les durées d'allaitement sont comprises « entre 6 et 12 mois » et « + de 12 mois ». Le quintile de richesse semble être associé positivement aux différentes durées d'allaitement car on constate une forte proportion d'allaitement « + de 12 mois » chez les mères appartenant au quintile moyen et au quintile le plus riche. Cependant, le sexe de l'enfant, le niveau d’éducation et les difficultés fonctionnelles de la mère ne semblent pas être associés au statut d'allaitement.

Le tableau IV présente les résultats de la régression logistique selon le statut d'allaitement afin de cerner au mieux l'influence des facteurs étudiés sur le maintien/arrêt de l'allaitement.

**Tableau IV:** Résultats de la régression logistique du statut d'allaitement en fonction des facteurs étudiés Results of logistic regression of breastfeeding status according to the factors studied

Statut d'allaitement	Moins de 6 mois	Plus de 6 mois	Odds ratios	IC à 95%	p
**Sexe de l'enfant**					
garçons	71,8 %	28,2 %	1		NS
filles	71,4 %	28,6 %	0,798	0,880-1,174	
**Zone d'habitat**					
urbain	72,8 %	27,2 %	1		< 0,05
rural	69,6 %	30,4 %	1,19	1,032-1.369	
**Espace de programmation territoriale (EPT)**					
EPT 1 : Nord-Centre	67,8 %	32,2 %	1,231	1,046-1,477	<0,05
EPT 2 : Nord-Est	73,8 %	26,2 %	0,849	0,572-1,023	NS
EPT 3 : Nord-Ouest	73,1 %	26,9 %	0,795	0,587-1,040	NS
EPT 4 : Hauts Plateaux-Centre	71,3 %	28,7 %	0,851	0,659-1,093	NS
EPT 5 : Hauts Plateaux-Est	66,2 %	33,8 %	1,485	1,123-1,677	<0,01
EPT 6 : Hauts Plateaux-Ouest	75,7 %	24,3 %	0,774	0,676-1,077	NS
EPT 7 : Sud	73,6 %	26,4 %	1		
**Niveau d'instruction de la mère**					
sans instruction	70,2 %	29,8 %	0,987	0,765-1,273	NS
primaire	70,5 %	29,5 %	0,974	0,757-1,251	
moyen	73,9 %	26,1 %	0,822	0,666-1,016	
secondaire	70,9 %	29,1 %	0,958	0,765-1,198	
supérieur	70,0 %	30,0 %	1		
**Difficulté fonctionnelle**					
à une difficulté fonctionnelle	71,6 %	28,4 %	0,988	0,665-1,495	NS
pas de difficulté fonctionnelle	71,5 %	28,5 %	1		
**Statut professionnel de la mère**					
à une profession	72,1 %	27,9 %	1		< 0,01
sans profession	67,5 %	32,5 %	1,489	1,107-1,947	
**Quintiles de l'indice de richesse**					
le plus pauvre	71,5 %	28,5 %	1		
le second	73,2 %	26,8 %	0,918	0,746-1,131	NS
le moyen	68,2 %	31,8 %	1,321	1,091-1,742	<0,05
le quatrième	75,1 %	24,9 %	1,061	0,861-1,307	NS
le plus riche	66,4 %	33,6 %	1,433	1,162-2,012	<0,05

Il ressort du tableau IV que les facteurs associés à l'allaitement de plus de six mois sont le milieu de résidence et la région géographique, le statut professionnel de la mère et le quintile de richesse. En effet, les femmes résidant dans les zones rurales ont presque 1,2 fois plus de chance d'allaiter plus de six mois par rapport à celles vivant dans les zones urbaines (OR= 1,19; IC [1,032-1,369]). Les mères résidant dans les régions géographiques du Nord-Centre et Hauts Plateaux-Est semblent avoir respectivement 1,2 et 1,5 fois plus de chance de maintenir l'allaitement au-delà de six mois en référence à celles vivant dans la région du Sud (OR = 1,23; IC [1,046-1,477]; OR = 1,48; IC [1,123-1,677]).

Les enfants des mères sans travail ont presque une fois et demie plus de chance d’être allaités après 6 mois que ceux des mères qui travaillent (OR = 1,489; IC [1,107-1,947]). Nos résultats révèlent également que l'appartenance des parents aux quintiles de richesse « moyen » et « le plus riche » semble augmenter la chance du maintien de l'allaitement après 6 mois avec respectivement 1,3 et 1,4 plus de chance en comparaison avec ceux relevant du quintile « le plus pauvre » (OR = 1,32; IC [1,091-1,742]; OR = 1,43; IC [1,162-2,012]). Cependant, les facteurs tels que le sexe de l'enfant, le niveau d'instruction et les difficultés fonctionnelles des mères ne semblent pas être des facteurs déterminants pour le maintien de l'allaitement après six mois.

## Discussion

Du point de vue de la mère, il est essentiel de ne pas instaurer de culpabilité autour du choix de l'allaitement. Les femmes peuvent avoir des raisons variées de choisir d'allaiter ou non, allant de considérations médicales et physiologiques à des facteurs pratiques et émotionnels. La société doit reconnaître la diversité des expériences maternelles et respecter les choix individuels, sans créer de pression inutile ou de jugement. Le respect de l'hygiène dans les soins apportés aux nourrissons constitue un aspect fondamental de la parentalité. D'un côté, les soins du mamelon impliquent des pratiques d'hygiène spécifiques, visant à assurer la santé du nourrisson tout en facilitant l'AM. De l'autre côté, la stérilisation des biberons est essentielle pour garantir la sécurité alimentaire des bébés nourris au lait en poudre.

Certains parents peuvent choisir de déléguer certaines tâches, y compris donner le biberon, à des membres de la famille ou à des personnes extérieures. Cette décision peut découler de divers facteurs tels que la disponibilité des parents, les exigences professionnelles ou même des choix personnels. Il est crucial de reconnaître que la parentalité ne repose pas uniquement sur les parents biologiques, mais peut également impliquer une collaboration avec d'autres membres de la famille ou des proches. Par ailleurs, certains aspects biologiques de l'allaitement, tels que la suppression de l'ovulation pendant la période d'allaitement intensif, peuvent avoir des implications sur la planification familiale. Cependant, ces aspects sont souvent méconnus ou mal compris.

Le choix entre l'AM et l'utilisation de biberons dépend de divers facteurs, et il est étroitement lié aux ressources, à l’éducation, ainsi qu’à des considérations socio-culturelles et religieuses. Les croyances et les normes culturelles peuvent influencer de manière significative les décisions des parents en matière d'alimentation infantile. L'exploitation des données de l'enquête MICS-6 nous a permis d'estimer la prévalence de l'AM en Algérie et les facteurs qui y sont associés. La prévalence de l'allaitement est quasiment identique à celle des études locales recensées. Cependant, les déterminants du recours à l'allaitement ne sont pas toujours en phase avec nos conclusions. Une étude antérieure montre que les mères d'enfants de sexe masculin optaient davantage pour l'allaitement que celles d'enfants de sexe féminin. Dans ce cas, elles font référence à l'influence du conjoint et à la place attribuée aux garçons dans la société [6, 11, 17, 20]. L'association significative entre le milieu de résidence et la durée de l'allaitement suggère que les femmes en zone rurale ont une probabilité plus élevée de maintenir l'allaitement au-delà de six mois. Cela pourrait être attribuable à des différences dans l'accès aux informations, aux ressources et aux normes culturelles. Ainsi, ce résultat corrobore celui de Korti *et al.,* qui ont affirmé que l'environnement urbain en Algérie est préjudiciable à l'allaitement [6, 10, 17, 20].

La différence de durée d'allaitement entre les régions géographiques, avec une probabilité plus élevée dans le Nord-Centre et les Hauts Plateaux-Est, met en évidence l'influence des facteurs locaux et régionaux. Les pratiques d'allaitement sont souvent profondément liées au contexte culturel. Les croyances, les valeurs familiales et les influences sociales propres à chaque région peuvent jouer un rôle déterminant. Des études complémentaires de nature socio-anthropologique pourraient explorer les déterminants locaux spécifiques, les attitudes culturelles envers l'allaitement.

La relation significative entre le statut professionnel de la mère et la durée de l'allaitement souligne l'impact potentiel des contraintes professionnelles sur les choix d'allaitement. Les mères sans emploi ont une probabilité plus élevée de maintenir l'allaitement, mettant en lumière les défis que les mères employées peuvent rencontrer en conciliant travail et allaitement. Cela suggère également la nécessité de politiques de soutien aux mères actives. Kadi *et al.* [9] ont montré qu'il n'existe pas de relation significative entre le statut professionnel de la mère et la pratique de l'allaitement. Nos résultats montrent plutôt le contraire. En effet, l'emploi de la mère est souvent considéré comme une contrainte pour l'initiation et/ou le maintien de l'allaitement. En Algérie, les femmes disposent d'un dispositif particulier leur permettant d'allaiter leurs enfants à raison de 2 heures par jour. Toutefois, ce ne sont pas toutes les femmes qui utilisent ce dispositif, surtout pour le cas de femmes exerçant des responsabilités professionnelles. Les parents appartenant aux quintiles de richesse « les plus riches » semblent augmenter les chances de maintien de l'AM après six mois. Cela pourrait refléter des différences dans l'accès aux ressources et les conditions socio-économiques qui influent sur les choix d'allaitement. Cette constatation est cohérente avec d'autres études qui ont souligné l'influence des facteurs économiques sur les pratiques d'allaitement. En effet, l’étude de Mecheri-Touati *et al*. a conclu que les mères d'un niveau socio-économique plus élevé pratiquent davantage l'AM [11]. Ces résultats sont en porte à faux avec le constat général du déclin de l'AM lié à l'amélioration du niveau de vie dans les pays en développement [5].

L'effet du niveau d’éducation de la mère sur le maintien de l'AM s'est avéré très controversé. Ainsi, nos résultats ne montrent aucune association entre le niveau d’éducation et le maintien de AM. Ce constat est en accord avec plusieurs études contextualisées menées dans des structures de santé de certaines wilayas [7, 3, 9]. En revanche, d'autres études ont abouti à une association positive entre l'AM et un faible niveau d’éducation maternelle. En effet, l’étude publiée par Korti *et al.* a montré qu’à 6 mois, le taux d'allaitement était trois fois plus élevé chez les femmes ayant un niveau d’éducation primaire par rapport à celles ayant un niveau plus élevé. L’étude de Mecheri-Touati *et al.* a révélé que ce sont les mères de niveau secondaire et supérieur qui allaitent plus longtemps. Les mères plus instruites semblent avoir une meilleure connaissance des avantages de l'AM. De plus, on s'attend à ce qu'elles prennent des décisions plus éclairées concernant leur propre santé et celle de leurs enfants.

Des études de nature qualitative ont mis en avant d'autres facteurs impactant le statut d'allaitement. Une recherche conduite dans le contexte algérien a relevé le manque d'information dans les centres de soins sur les modes d'allaitement et l'insuffisance des connaissances sur les techniques de pratique de l'allaitement chez les jeunes mères. 58 % d'entre elles s'en remettent à leur famille (mère, belle-mère, mari et sœur) pour prendre les décisions concernant le choix de l'allaitement [4].

Les corrélations identifiées soulignent la complexité des déterminants de la durée de l'allaitement en Algérie. Cette compréhension plus fine des influences peut orienter les interventions et les politiques visant à promouvoir et à soutenir l'AM au-delà des six premiers mois. Il est important de noter que ces corrélations ne déterminent pas une relation causale directe, mais soulignent plutôt des associations significatives qui méritent une exploration plus approfondie dans le contexte spécifique de l'Algérie.

Notre recherche est le premier travail qui a exploité un échantillon représentatif de la population générale représentant les 7 espaces de programmation territoriale, contrairement à toutes les études menées en Algérie où la taille des échantillons était réduite et ne concernait qu'une wilaya. Ceci rend nos résultats plus robustes et plus pertinents. Quant aux limites de cette étude, elles résident dans le nombre réduit de variables prédictives contenues dans la base de données.

Un bon nombre de variables pertinentes qui déterminent le recours à l'AM ont été identifiées dans la littérature [14, 15, 16, 24]. En outre, d'autres limitations comprennent le caractère rétrospectif des réponses des participants, ce qui peut causer certaines difficultés en termes d'exactitude des réponses. Il convient de noter que les variables prises en considération dans cette étude ne peuvent expliquer qu'une partie des déterminants du maintien de l'AM au-delà de 6 mois. Par conséquent, d'autres facteurs socio-anthropologiques peuvent être explorés pour mieux comprendre le phénomène étudié. La mise en perspective des résultats de l’étude algérienne avec des recherches menées dans des contextes internationaux souligne à la fois la variabilité et l'universalité des déterminants de la durée de l'allaitement [21, 22, 23]. Les similitudes observées dans certains résultats suggèrent des tendances communes, mais les divergences soulignent l'importance de prendre en compte les spécificités culturelles, économiques et sociales propres à chaque contexte. Ces observations renforcent la nécessité d'approches personnalisées pour promouvoir et soutenir l'AM en tenant compte des particularités locales.

## Conclusion

Notre étude a montré que la durée de l'allaitement varie en fonction de la zone de résidence, de la région géographique, du statut professionnel de la mère et du quintile de richesse. Par exemple, les mères vivant en milieu rural, dans certaines régions géographiques, celles sans emploi et celles appartenant au quintile de richesse moyen ou élevé ont une probabilité plus élevée de maintenir l'allaitement pendant plus de six mois. Cependant, le sexe de l'enfant, le niveau d’éducation de la mère et les difficultés fonctionnelles n'ont pas semblé exercer une influence significative sur la durée de l'allaitement au-delà de six mois.

Néanmoins, malgré de nombreuses études visant à mieux comprendre le comportement des mères en matière d'allaitement et diverses initiatives visant à le promouvoir, de nombreux pays présentent des taux assez faibles. Par conséquent, la tendance à la baisse de l'AM est directement liée aux progrès dans la production et la commercialisation des laits industriels, au manque d'informations et de sensibilisation parmi les mères et au manque de formation des professionnels de la santé. À cet égard, il est fortement recommandé d'encourager les changements de comportement et d'améliorer la communication sur la durée de l'AM, d'augmenter l'utilisation de conseils postnataux et de former le personnel paramédical conformément aux recommandations, avec un soutien aux mères. En définitive, ce travail doit être enrichi par des études complémentaires de nature qualitative sur les facteurs de l'abandon précoce de l'AM.

## Contribution des auteurs

Ce travail a été réalisé conjointement par les deux auteurs. AZ a conçu l’étude, effectué la recherche documentaire, rédigé et révisé l'article. ASY a réalisé les analyses statistiques et révisé le document. Les deux auteurs ont lu et approuvé le manuscrit final.

## Liens d'intérêts

Les auteurs déclarent ne pas avoir d'intérêt direct ou indirect (financier ou en nature) avec un organisme privé, industriel ou commercial en relation avec le présent article.
